# Association of Participation in Warm-Up Exercises with Complications, Subsequent Injury Frequency, and Recovery Duration Among Athletes with a History of Injury: A Physical Activity Epidemiology Study Using Secondary Survey Data

**DOI:** 10.3390/medicina62040719

**Published:** 2026-04-09

**Authors:** Eun-Hee Park, Daekeun Kwon, Jeonga Kwon

**Affiliations:** 1 Department of Sports Leisure, Sungshin Women’s University, Seongbuk-gu, Seoul 02844, Republic of Korea; peh7904@gmail.com; 2 Institute of Sports Health Science, Sunmoon University, Asan 31460, Republic of Korea; 3 Department of Elementary Education, College of First, Korea National University of Education, Cheongju 28173, Republic of Korea

**Keywords:** adverse outcomes, exercise safety incidents, follow-up injury incidence, preparatory exercise, rehabilitation period, sports injury

## Abstract

*Background and Objectives*: Engaging in sports activities presents several benefits but also carries a risk of injury. Performing warm-up exercises may serve as a preventive measure against sports injuries. This study explored how participating in warm-up exercises is associated with complications, subsequent injury frequency, and recovery duration among athletes with a history of sports injuries. *Materials and Methods*: In this study, we performed cross-sectional secondary analysis of data derived from the 2024 Sports Safety Accident Survey conducted by the Korea Sports Safety Foundation, which is a nationally administered survey based on a structured questionnaire. The survey, conducted from November 2024 to December 2024, targeted 10,000 athletes aged ≥12 years registered in 64 sports nationwide. We utilized the data of 6063 athletes who had experienced sports injuries. The collected data were analyzed using frequency analyses, chi-squared tests, and multivariate logistic regression analyses. This study was conducted as a physical activity epidemiology study to examine associations between warm-up exercise participation and injury outcomes. *Results*: The likelihood of complications was higher among those who responded “not really” or “neutral” regarding participation in warm-up exercises. The likelihood of injuries was higher among those who responded “neutral” or “somewhat”. Furthermore, the likelihood of prolonged recovery was higher among those who responded “not really”, “neutral”, or “somewhat”. *Conclusions*: Performing warm-up exercises is an essential measure for athletes with a history of injuries to safely engage in sports activities. Therefore, it is necessary to highlight the importance of warm-up exercises among athletes and to implement a safety management system to encourage consistent performance.

## 1. Introduction

In contemporary life, excessive academic and work burdens make it difficult to adequately care for one’s health. These factors not only deteriorate physical health but also increase mental stress. In this context, participation in sports activities offers various benefits, such as health promotion, improvement in physical fitness, psychological stability, and enhancement of social interactions. Regarding physical health, engaging in sports activities enhances cardiopulmonary function, prevents obesity, and increases muscle strength, all of which reduce the incidence of cardiovascular diseases, cancer, and mortality rates [1]. Regarding mental health, participating in sports activities increases self-esteem, reduces stress, decreases suicidal impulses, lowers the incidence of depression and anxiety, and promotes social development by bolstering social engagement, a sense of achievement, and identity formation [2,3]. In other words, participating in sports activities can help individuals restore their health and improve quality of life.

Although participation in sports activities presents several benefits, it also carries the risk of injuries, such as contusions, hematomas, dislocations, subluxations, and muscle, ligament, and tendon ruptures [4,5]. Moreover, these injuries can transcend physical damage and cause lasting complications. Research has shown that individuals with a history of injuries can experience physical sequelae such as chronic pain and decreased joint or muscle function, which can result in physical disabilities and reduced athletic performance [6,7,8,9]. Bone-related sports injuries can cause menstrual irregularities in female athletes [7]. Athletes who have experienced sports-related concussions are more likely to sustain musculoskeletal injuries [8]. Furthermore, most boxers suffer dentofacial injuries and endodontic sequelae [9]. Sports injuries can also induce emotional complications, such as anxiety, depression, decreased self-confidence, and suicide attempts [10,11,12]. Chow et al. [11] conducted a meta-analysis on sports injuries, mental health, and well-being. They reported that the occurrence of concussions is strongly associated with impaired mental health and well-being. Additionally, Lichtenstein et al. [12] found that athletes who have experienced musculoskeletal injuries report relatively higher levels of stress and depression than those who have not experienced such injuries. These findings suggest that sports injuries can go beyond physical damage and cause long-term decline in physical function, mental health, and even quality of life.

Individuals with a history of sports injuries face the risk of experiencing not only complications but also re-injury at the same site. Additionally, their recovery period tends to be longer. Numerous studies have reported that injured areas can be re-injured during sports activities, and re-injuries have longer return-to-play durations and worse conditions than new injuries [13,14,15]. Among professional and elite athletes, the recurrence rate of injury sites varies based on sport, ranging from 5% to 21% [13]. Among 10–20-year-old track and field athletes affiliated with the French Athletics Federation, half of those who had experienced injuries reported a re-injury at the same site, and one-third experienced sequelae [14]. In Welton et al.’s [15] study, 10.5% of the injuries among US high school athletes were re-injuries, and these athletes experienced longer return-to-play durations and greater likelihood of surgery than those with new injuries. Therefore, in order to play safely and maintain long-term health and performance, it is important to prevent not only injuries but also re-injuries, and systematic recovery management is essential.

The treatment of sports injuries can be time-consuming, difficult, and expensive; therefore, preventive strategies are warranted [6]. Warm-up exercises can be considered a fundamental measure in preventing sports injuries. These exercises increase muscle temperature, boost blood flow, expand the range of motion in joints, and help alleviate the burden on the cardiovascular system [16]. Numerous studies have indicated that warm-up exercises effectively reduce the risk of sports injuries. Ding et al. [17] conducted a meta-analysis on the role of warm-up exercises in preventing sports injuries among children and adolescents. The results confirmed that warm-up programs for injury prevention significantly reduce the incidence of sports injuries among children and adolescents [17]. Chen et al. [18] reported that warm-up exercises have a significant impact on the explosive power and handball throwing velocity of elite male university handball players. Furthermore, Afonso et al. [19] reported that warm-up exercises enhance exercise performance, suggesting that they induce neuromuscular activation or potentiation, thus preparing the body for the demands of the training session. They also suggested that warm-up exercises mentally prepare the athletes for upcoming tasks, shifting their focus from everyday concerns to the demands of the sport [19].

Individuals who have experienced sports injuries often suffer from complications, re-injuries, and longer recovery periods. This makes it vital to explore whether participation in warm-up exercises helps prevent complications, re-injuries, and longer recovery periods. Therefore, this study examined how participation in warm-up exercises is related to complications, subsequent injury frequency, and recovery duration among athletes with a history of sports injuries. We hypothesized that performing warm-up exercises is strongly associated with reduced complications, lower frequency of injuries, and shorter recovery durations among athletes who have experienced sports injuries. The findings of this study are expected to serve as fundamental data for effective injury prevention and management. Additionally, they may facilitate safer participation in sports activities and help create a healthier society.

## 2. Materials and Methods

### 2.1. Design, Data, and Study Population

This study was conducted using data from the 2024 Sports Safety Accident Survey conducted by the Korea Sports Safety Foundation, which is a nationally administered survey using a structured questionnaire [20,21]. The Sports Safety Accident Survey, which is based on a cross-sectional physical activity epidemiology study identifies the status and causes of sports accidents among recreational and professional athletes. The 2024 Sports Safety Accident Survey was designed by updating the survey items and questionnaire frameworks developed in 2015 and 2019. The survey instrument was previously validated in the 2015 and 2019 surveys, and the core items demonstrated acceptable reliability. The participants were proportionally sampled based on the type of athlete, with separate sampling for recreational and professional athletes. A participant list was constructed, and online surveys were conducted with the individuals on this list. Potential sources of bias include self-reported injury history, recall bias, which were noted as limitations. The 2024 Sports Safety Accident Survey was conducted with 10,000 athletes aged 12 or older across 64 sports registered with the Korea Sports Council, which oversees sports administration in Korea. Data from participants with missing or incomplete data for key variables were excluded from the analysis after deletion. In other words, participants must be 12 years of age or older and must be registered in 64 sports overseen by the Korea Sports Council. The Korea Sports Safety Foundation obtained consent regarding personal data protection and confidentiality before conducting the survey. After the survey, it removed personally identifiable information and shared raw data on their website (available at https://www.sportsafety.or.kr/front/board/boardContentsListPage.do?board_id=42 (accessed on 1 January 2026)). We downloaded this data for use in our study. Among the 10,000 survey participants, we used the data of 6063 athletes who had experienced sports injuries relevant to the purpose of this study, whereas data for the remaining 3937 individuals with no experience of sports injuries were excluded. Given that this study utilized a publicly available dataset that did not contain personally identifiable information such as home addresses, telephone numbers, and social security numbers. Instead, they contain ID numbers. All research procedures were approved by the Korea Sports Safety Foundation (approval number: 93-2024; date: 1 January 2024), and this study was conducted according to the principles outlined in the Declaration of Helsinki.

### 2.2. Measures

#### 2.2.1. Independent Variable

The independent variable, participation in warm-up exercises, was measured using the question, “Do you perform warm-up exercises before exercising?” The response options were “not at all”, “not really”, “neutral”, “somewhat”, and “very much”. Although this variable has a natural order, it was treated as categorical to avoid assuming a linear relationship and to allow for potential non-linear associations. Given the possibility of a threshold effect, where significant protective benefits may occur only at high levels of adherence, categorizing participants allows for identification of potential non-linear patterns in injury prevention outcomes across different adherence levels.

#### 2.2.2. Dependent Variables

The dependent variables were the occurrence of complications, subsequent injury frequency and recovery duration. The occurrence of complications was determined using the question, “Did you experience any sequelae due to injury?” The response options were “yes” and “no”. Subsequent injury frequency was measured using the question, “How many times did you experience sports injuries over the past year?” Participants were asked to indicate their subsequent injury frequency in numerical form. For this study, we categorized the responses as “1”, “2”, “3”, “4”, and “5 or more”. 5 or more was selected as the reference category to facilitate interpretation of the odds of experiencing fewer injuries compared with the highest frequency group. The recovery duration was assessed using the question, “How long did it take you to fully recover and return to your sports activities?” The response options were “not much time; resumed activities immediately”, “less than a week”, “1–2 weeks”, “3–4 weeks”, “5–8 weeks”, and “9 or more weeks”.

#### 2.2.3. Covariate Variables

The covariate variables were athlete type, sex, age, frequency of sports participation, participation in a sports club, and injury recurrence. Athletes were classified as either recreational athletes or professional athletes. Sex was classified as male or female. Age was categorized as “13–18”, “19–29”, “30–39”, “40–49”, “50–59”, “60–64”, and “65 or older”. Frequency of sports participation was measured using the question, “How often did you participate in sports activities over the past year?” The response options were “daily”, “4–6 times a week”, “2–3 times a week”, “once a week”, “2–3 times a month”, “once a month”, “once every 2–3 months”, “once every 4–5 months”, “once every 6 months”, and “not regularly”. Participation in a sports club was determined by asking, “Are you a member of a sports club?” The response options were “yes” and “no”. Injury recurrence was determined using the question, “Have you ever experienced the same type of injury as a previous serious one?” The response options were “yes” and “no”. The responses for these variables were used without any modifications.

### 2.3. Data Analysis

All statistical analyses were performed using SPSS for Windows (version 23.0; IBM Corp., Armonk, NY, USA). First, we conducted a frequency analysis on participant characteristics: athlete type, sex, age, frequency of sports participation, participation in a sports club, injury recurrence, occurrence of complications, subsequent injury frequency, and recovery duration. Second, we conducted chi-squared tests to identify differences in participation in warm-up exercises based on participant characteristics. Third, we performed multivariate logistic regression analyses to analyze how participation in warm-up exercises is associated with the occurrence of complications, subsequent injury frequency, and recovery duration. Binary logistic regression was used for the “complications” outcome, and multinomial logistic regression was used for the nominal outcomes “subsequent injury frequency” and “recovery duration”. All models were adjusted for athlete type, sex, age, frequency of sports participation, sports club membership, and injury recurrence. Odds ratios (ORs), 95% confidence intervals (CIs), and *p*-values were determined. Statistical significance was set at *p* < 0.05.

## 3. Results

### 3.1. Participant Characteristics

As shown in Table 1, 0.7% of the participants reported “not at all”, 4.1% reported “not really”, 16.3% reported “neutral”, 47.9% reported “somewhat”, and 31.0% reported “very much” regarding participation in warm-up exercises. Regarding sex, 69.4% of the participants were male, while 30.6% were female. Regarding age, 27.0% of the participants were 13–18 years old, 17.0% were 19–29 years old, 20.2% were 30–39 years old, 19.3% were 40–49 years old, 11.6% were 50–59 years old, 2.9% were 60–64 years old, and 2.0% were 65 years old or older. Individuals who engaged in sports activities 4–6 times a week were the highest in number (24.8%). Most participants were not part of a sports club (56.7%). A majority of the participants reported injury recurrence (77.2%) and the absence of complications (73.0%). Regarding subsequent injury frequency, 35.5% of the participants reported once, 28.4% reported twice, 15.9% reported three times, 4.9% reported four times, and 15.3% reported five times or more. Regarding recovery duration, 15.2% of the participants reported less than a week, 29.1% reported 1–2 weeks, 22.0% reported 3–4 weeks, 8.7% reported 5–8 weeks, and 9.2% reported 9 or more weeks. The rest reported that they resumed activities immediately.

### 3.2. Differences in Participation in Warm-Up Exercises Based on Participant Characteristics

Table 2 presents the results of identifying differences in participation in warm-up exercises based on participant characteristics. Participation in warm-up exercises differed significantly based on the athlete type (χ2 = 866.537, *p* < 0.001), sex (χ2 = 10.302, *p* = 0.036), age (χ2 = 1150.945, *p* < 0.001), frequency of sports participation (χ2 = 1166.836, *p* < 0.001), participation in a sports club (χ2 = 70.711, *p* < 0.001), injury recurrence (χ2 = 106.663, *p* < 0.001), subsequent injury frequency (χ2 = 151.753, *p* < 0.001), and recovery duration (χ2 = 147.584, *p* < 0.001).

### 3.3. Association Between Participation in Warm-Up Exercises and the Occurrence of Complications

Table 3 shows the results of analyzing the association between participation in warm-up exercises and the occurrence of complications. The OR for experiencing complications was 1.650 (95% CI: 1.191–2.287, *p* = 0.003) among participants who responded “not really” regarding participation in warm-up exercises. The OR was 1.319 (95% CI: 1.075–1.620, *p* = 0.008) among those who responded “neutral”. These findings indicated that the likelihood of complications is higher among those who responded “not really” or “neutral”. In other words, actively performing warm-up exercises reduced the likelihood of complications following an injury.

### 3.4. Association Between Participation in Warm-Up Exercises and Subsequent Injury Frequency

Table 4 presents the results of identifying the association between participation in warm-up exercises and subsequent injury frequency. The OR for experiencing sports injuries once was 1.330 (95% CI: 1.007–1.758, *p* = 0.045) among those who responded “neutral” regarding participating in warm-up exercises and 1.332 (95% CI: 1.096–1.619, *p* = 0.004) among those who responded “somewhat”. The OR for experiencing sports injuries two times was 1.441 (95% CI: 1.085–1.914, *p* = 0.012) among those who responded “neutral” and 1.406 (95% CI: 1.151–1.717, *p* = 0.001) among those who responded “somewhat”. These findings indicate that the likelihood of sports injuries is higher among those who responded “neutral” or “somewhat”. In other words, actively performing warm-up exercises reduced the likelihood of sports injuries.

### 3.5. Association Between Participation in Warm-Up Exercises and Recovery Duration

Table 5 presents the results of identifying the association between participation in warm-up exercises and recovery duration. The OR for immediate resumption of sports activities was 3.560 (95% CI: 1.743–7.272, *p* < 0.001) among those who responded “not really” regarding participating in warm-up exercises, 1.940 (95% CI: 1.323–2.843, *p* = 0.001) among those who responded “neutral”, and 1.766 (95% CI: 1.350–2.310, *p* < 0.001) among those who responded “somewhat”. The OR for recovery in less than a week was 4.464 (95% CI: 2.243–8.885, *p* < 0.001) among those who responded “not really”, 2.639 (95% CI: 1.824–3.816, *p* < 0.001) among those who responded “neutral”, and 1.694 (95% CI: 1.291–2.221, *p* < 0.001) among those who responded “somewhat”. The OR for recovery in 1–2 weeks was 2.743 (95% CI: 1.419–5.305, *p* = 0.003) among those who responded “not really”, 2.010 (95% CI: 1.432–2.823, *p* < 0.001) among those who responded “neutral”, and 1.834 (95% CI: 1.440–2.335, *p* < 0.001) among those who responded “somewhat”. The OR for recovery in 3–4 weeks was 2.193 (95% CI: 1.121–4.292, *p* = 0.022) among those who responded “not really”, 1.530 (95% CI: 1.079–2.167, *p* = 0.017) among those who responded “neutral”, and 1.634 (95% CI: 1.278–2.089, *p* < 0.001) among those who responded “somewhat”. These findings indicated that the likelihood of prolonged recovery is higher among those who responded “not really”, “neutral”, or “somewhat”. In other words, actively performing warm-up exercises reduced the likelihood of prolonged recovery.

## 4. Discussion

### 4.1. Interpretation of the Findings

Our results showed that 78.9% of the participants responded positively to participating in warm-up exercises. However, 21.1% did not actively participate in warm-up exercises, with lower participation among recreational athletes. Participation in warm-up exercises helps prevent injuries by increasing body temperature and joint range of motion. Additionally, it improves athletic performance [16,17,18,19]. Although a significant number of participants performed warm-up exercises, recreational athletes remain exposed to the risk of sports injury. Therefore, it is necessary to emphasize the importance of warm-up exercises, even in recreational sports.

However, although these associations were found to be statistically significant, the magnitude of the effects was moderate rather than substantial, thereby indicating that warm-up exercises may represent a contributing factor rather than the sole determinant of post-injury outcomes. These findings thus indicate that compared with those who actively performed warm-ups, participants with lower engagement in warm-up exercises had a greater likelihood of experiencing complications. Accordingly, our findings indicate that actively performing warm-up exercises reduces the likelihood of complications following injury, which is consistent with previous research that has shown that warm-up exercises contribute to preventing complications [6,17,22]. Ding et al. [17] reported that warm-up interventions help reduce complications such as muscle weakness among children and adolescent athletes. In Herman et al.’s [22] study, a warm-up program reduced the incidence of pain among young amateur female soccer players, partially aligning with the results of this study. These results suggest that, among athletes with a history of injury, warm-up exercises activate or stimulate the muscles and ligaments around the affected areas, reducing instability and improving motor control, thus lowering the likelihood of repeated injuries due to complications [22]. However, previous studies have emphasized that warm-up exercises should be designed as structured programs specifically aimed at injury prevention and tailored to the demands of the sport [6,17,22]. For example, Ding et al. [17] recommended implementing warm-up exercises through comprehensive programs, neuromuscular programs, or balance programs to prevent sports injuries among children and adolescents. Therefore, it can be concluded that a systematic warm-up program can reduce complications in athletes with a history of injury. Such programs can also contribute to the prevention of repetitive injuries and stable recovery.

Third, our results show that actively performing warm-up exercises reduces the likelihood of injury. This finding aligns with those of previous studies demonstrating that warm-up exercises help decrease the frequency of injuries [23,24,25]. Soligard et al. [23] reported that participation in warm-up exercises helps reduce injury rates, particularly those of severe injuries, among young female soccer players. Paravlic et al. [24] reported significant reductions in the incidence of injury in a group of basketball players who performed warm-up exercises. Verhagen et al. [25] reported that participation in warm-up exercises is strongly associated with reduced injuries, particularly moderate ones, among young volleyball players. Collectively, these findings indicate that although ORs provide evidence of moderate effects, the consistent protective trend identified in multiple sports highlights the practical relevance of warm-up routines in reducing the risk of injury. The strong correlation between performing warm-up exercises and reduced injury rates is likely because athletes with a history of injury face a risk of re-injury, which can be mitigated through warm-up routines [26]. These athletes can enhance strength, balance, and mobility through warm-up exercises, aiding recovery and reducing injury incidence during matches and training [27]. These findings suggest that warm-up exercises are effective in preventing injuries. Therefore, warm-up exercises can be considered a key means of reducing the risk of injury in athletes. The ORs in this study provide quantitative support for these associations, indicating that regular participation in warm-up exercises is related to a statistically significant reduction in complications, subsequent injury frequency, and recovery duration. These findings are broadly consistent with prior epidemiological studies examining structured injury prevention programs and sports warm-ups, which report similar protective effects [6,17,23].

Fourth, our results indicate that actively performing warm-up exercises reduces the likelihood of prolonged recovery. Warm-up exercises increase blood flow around the muscles and joints, helping the injured area recover and reducing the risk of re-injury, thereby potentially shortening the treatment period after injury [28]. Sadigursky et al. [29] reported that participation in warm-up programs significantly reduces injury rates among soccer players and that the less severe the injury, the shorter the recovery period. This finding partially aligns with the findings of this study. Koubaa et al. [30] emphasized that warm-up exercises help reduce oxidative stress and muscle damage after exercise. Given that a reduction in oxidative stress is closely related to injury recovery, reducing muscle damage through warm-up exercises can ultimately shorten the treatment period [30]. By establishing an association between the physiological mechanisms and observed ORs, we highlight a coherent link between regular warm-up and reduced complications and accelerated recovery. Therefore, performing warm-up exercises regularly is beneficial for both treatment effectiveness and prevention of re-injury. However, as this study employed a cross-sectional design, causal relationships between warm-up exercises and injury outcomes cannot be established. Additionally, reliance on self-reported data introduces potential recall bias, which may affect the accuracy of the reported frequency of warm-up participation and injury outcomes. These limitations should be considered when interpreting the findings.

### 4.2. Practical Implications of the Findings

Among athletes with a history of injury, 21.1% did not actively participate in warm-up exercises. This finding reflects not only non-engagement in warm-up activities but also a lack of awareness about the risks associated with sports activities and appropriate safety measures. This emphasizes the need to highlight the importance of warm-up exercises, increase awareness, and develop safety management systems among individuals who participate in sports activities.

In this study, recreational athletes showed particularly low participation in warm-up exercises. This finding underscores the need to increase awareness about sports safety and promote warm-up exercises among recreational athletes. Unlike professional athletes, recreational athletes engage in sports activities out of their own volition [31,32]. Additionally, in recreational sports, safety-centric oversight [33] and injury-related education is generally inadequate [20]. This is because recreational sports instructors tend to focus on member management or coaching skills and lack safety-related expertise, or a safety education system is not in place [34,35]. The recreational sports environment plays a decisive role in whether participants view warm-up exercises as a “choice” or a “necessity” [20,31,32,33,34,35]. Therefore, in recreational sports settings, it is necessary to enhance instructors’ expertise in sports safety education and provide proper education on warm-up exercises, injuries that frequently occur in sports, and injury prevention measures.

Compared to professional sports, where sports safety manuals and systems are present, recreational sports lack a comprehensive safety management system [36]. This is due to a combination of factors, including the voluntary nature of participation, the lack of expertise among professionals [34], and the absence of standardized safety policies [37]. Therefore, it is necessary to establish a safety management system, provide systematic safety education, and prevent and supervise sports-related accidents in recreational sports settings. Practical steps to achieve this may include incorporating short, structured warm-up routines into all group activities, providing instructors with brief training on injury prevention and warm-up techniques, and using simple monitoring or feedback systems to encourage participant engagement [6,17,23]. These measures are low-cost, evidence-based, and can be easily integrated into existing recreational programs, helping to reduce injuries and improve safety awareness [6,17,23]. Overall, implementing these strategies can foster a safer and more effective recreational sports environment, ensuring that participants benefit from the physical and psychological advantages of exercise while minimizing the risk of injury.

### 4.3. Limitations

This study has several limitations. First, warm-up exercises differ based on the sport and range from bodyweight exercises, dynamic stretching, to aerobic exercises [38]. As this study focused on the extent to which participants performed warm-up exercises, it did not consider which type of warm-up exercises were performed. Second, data concerning the performance of warm-up exercises, the occurrence of complications, subsequent injury frequency, and recovery duration were based on subjective judgment rather than scientific measurements. Third, multivariate logistic regression was useful in this study; however, given that warm-up exercises and injury occurrence can involve many interacting and non-linear factors, the regression method may not have fully leveraged the potential of the large dataset used in this study. Future studies should utilize structural equation modeling or hierarchical linear modeling. Fourth, this study is limited by potential selection bias in the choice of variables and the lack of causal evidence stemming from raw data in secondary research. Additionally, the cross-sectional nature of the study and reliance on self-reported measures further restrict the ability to draw causal inferences and may have introduced recall bias, which aligns with the considerations noted in the main findings. Future studies should conduct experimental research to clarify causal relationships.

## 5. Conclusions

Among athletes with a history of injuries, a considerable number do not regularly perform warm-up exercises, with participation especially low among recreational athletes. Our results show an association between performing warm-up exercises and a lower likelihood of complications, fewer repeated injuries, and faster recovery times. Because this study is cross-sectional, we cannot say for certain that warm-up exercises directly cause these benefits, but the findings suggest that regular warm-ups may play an important role in supporting athlete safety and recovery. These findings highlight the need for better education and support in recreational sports settings, where guidance on injury prevention is often limited. Providing athletes and instructors with practical advice on warm-up routines, along with simple monitoring or feedback systems, could help increase participation, reduce injuries, and improve recovery. Overall, encouraging regular warm-up exercises is a low-cost, evidence-based way to make sports safer and more effective, allowing athletes—especially those in recreational sports—to enjoy the benefits of exercise while lowering their risk of injury.

## Figures and Tables

**Table 1:** Participant characteristics (n = 6063).

Characteristic	Categories	n (%)
Participation in warm-up exercises	Not at all	40 (0.7%)
	Not really	248 (4.1%)
	Neutral	987 (16.3%)
	Somewhat	2905 (47.9%)
	Very much	1883 (31.0%)
Type of athlete	Recreational athlete	3442 (56.8%)
	Professional athlete	2621 (43.2%)
Sex	Male	4210 (69.4%)
	Female	1853 (30.6%)
Age	13–18	1640 (27.0%)
	19–29	1032 (17.0%)
	30–39	1225 (20.2%)
	40–49	1168 (19.3%)
	50–59	706 (11.6%)
	60–64	173 (2.9%)
	65 or older	119 (2.0%)
Frequency of sports participation	Daily	953 (15.7%)
	4–6 times a week	1501 (24.8%)
	2–3 times a week	1309 (21.6%)
	Once a week	1034 (17.1%)
	2–3 times a month	622 (10.3%)
	Once a month	315 (5.2%)
	Once every 2–3 months	141 (2.3%)
	Once every 4–5 months	31 (0.5%)
	Once every 6 months	39 (0.6%)
	Not regularly	118 (1.9%)
Participation in a sports club	Yes	2628 (43.3%)
	No	3435 (56.7%)
Injury recurrence	Yes	4682 (77.2%)
	No	1381 (22.8%)
Occurrence of complications	Yes	1638 (27.0%)
	No	4425 (73.0%)
Subsequent injury frequency	1	2153 (35.5%)
	2	1724 (28.4%)
	3	966 (15.9%)
	4	295 (4.9%)
	5 or more	925 (15.3%)
Recovery duration	Not much time; resumed activities immediately	961 (15.8%)
	Less than a week	922 (15.2%)
	1–2 weeks	1763 (29.1%)
	3–4 weeks	1334 (22.0%)
	5–8 weeks	525 (8.7%)
	9 or more weeks	558 (9.2%)

**Table 2:** Differences in participation in warm-up exercises based on participant characteristics (n = 6063).

Characteristic		Categories	Participation in Warm-Up Exercises					χ2 (P)
			Not at All	Not Really	Neutral	Somewhat	Very Much	
Covariate variables	Type of athlete	Recreational athlete	24 (60.0%)	184 (74.2%)	725 (73.5%)	1961 (67.5%)	548 (29.1%)	866.537 (<0.001 ***)
		Professional athlete	16 (40.0%)	64 (25.8%)	262 (26.5%)	944 (32.5%)	1335 (70.9%)	
	Sex	Male	22 (55.0%)	167 (67.3%)	682 (69.1%)	1989 (68.5%)	1350 (71.7%)	10.302 (0.036 *)
		Female	18 (45.0%)	81 (32.7%)	305 (30.9%)	916 (31.5%)	533 (28.3%)	
	Age	13–18	4 (10.0%)	15 (6.0%)	91 (9.2%)	515 (17.7%)	1015 (53.9%)	1150.945 (<0.001 ***)
		19–29	7 (17.5%)	41 (16.5%)	160 (16.2%)	503 (17.3%)	321 (17.1%)	
		30–39	13 (32.5%)	69 (27.8%)	233 (23.6%)	674 (23.2%)	236 (12.5%)	
		40–49	9 (22.5%)	67 (27.1%)	262 (26.5%)	659 (22.8%)	171 (9.1%)	
		50–59	4 (10.0%)	37 (14.9%)	182 (18.5%)	390 (13.4%)	93 (4.9%)	
		60–64	2 (5.0%)	10 (4.1%)	33 (3.4%)	96 (3.3%)	32 (1.7%)	
		65 or older	1 (2.5%)	9 (3.6%)	26 (2.6%)	68 (2.3%)	15 (0.8%)	
	Frequency of sports participation	Daily	5 (12.5%)	15 (6.0%)	77 (7.8%)	279 (9.6%)	577 (30.6%)	1166.836 (<0.001 ***)
		4–6 times a week	8 (20.0%)	31 (12.5%)	134 (13.6%)	587 (20.2%)	741 (39.4%)	
		2–3 times a week	5 (12.5%)	51 (20.6%)	257 (26.0%)	742 (25.5%)	254 (13.4%)	
		Once a week	9 (22.5%)	63 (25.5%)	197 (20.0%)	609 (21.0%)	156 (8.3%)	
		2–3 times a month	5 (12.5%)	36 (14.5%)	157 (15.9%)	349 (12.0%)	75 (4.0%)	
		Once a month	1 (2.5%)	19 (7.7%)	68 (6.9%)	191 (6.6%)	36 (1.9%)	
		Once every 2–3 months	2 (5.0%)	13 (5.2%)	36 (3.6%)	64 (2.2%)	26 (1.4%)	
		Once every 4–5 months	2 (5.0%)	2 (0.8%)	6 (0.6%)	19 (0.7%)	2 (0.1%)	
		Once every 6 months	0 (0.0%)	4 (1.6%)	13 (1.3%)	19 (0.7%)	3 (0.2%)	
		Not regularly	3 (7.5%)	14 (5.6%)	42 (4.3%)	46 (1.5%)	13 (0.7%)	
	Participation in a sports club	Yes	17 (42.5%)	100 (40.3%)	394 (39.9%)	1418 (48.8%)	699 (37.1%)	70.711 (<0.001 ***)
		No	23 (57.5%)	148 (59.7%)	593 (60.1%)	1487 (51.2%)	1184 (62.9%)	
	Injury recurrence	Yes	24 (60.0%)	177 (71.4%)	703 (71.2%)	2176 (74.9%)	1602 (85.1%)	106.663 (<0.001 ***)
		No	16 (40.0%)	71 (28.6%)	284 (28.8%)	729 (25.1%)	281 (14.9%)	
Dependent variables	Occurrence of complications	Yes	10 (25.0%)	76 (30.6%)	275 (27.9%)	773 (26.6%)	504 (26.8%)	2.401 (0.662)
		No	30 (75.0%)	172 (69.4%)	712 (72.1%)	2132 (73.4%)	1379 (73.2%)	
	Subsequent injury frequency	1	15 (37.5%)	100 (40.3%)	385 (39.0%)	1083 (37.3%)	570 (30.2%)	151.753 (<0.001 ***)
		2	11 (27.5%)	74 (29.8%)	304 (30.8%)	884 (30.4%)	451 (24.0%)	
		3	5 (12.5%)	37 (14.9%)	151 (15.3%)	454 (15.6%)	319 (16.9%)	
		4	2 (5.0%)	7 (2.9%)	43 (4.4%)	119 (4.1%)	124 (6.6%)	
		5 or more	7 (17.5%)	30 (12.1%)	104 (10.5%)	365 (12.6%)	419 (22.3%)	
	Recovery duration	Not much time; resumed activities immediately	8 (20.0%)	40 (16.1%)	137 (13.9%)	441 (15.2%)	335 (17.8%)	147.584 (<0.001 ***)
		Less than a week	8 (20.0%)	53 (21.4%)	192 (19.5%)	408 (14.0%)	261 (13.9%)	
		1–2 weeks	8 (20.0%)	72 (29.0%)	314 (31.8%)	916 (31.5%)	453 (24.1%)	
		3–4 weeks	4 (10.0%)	51 (20.6%)	206 (20.8%)	693 (23.9%)	380 (20.2%)	
		5–8 weeks	7 (17.5%)	20 (8.1%)	71 (7.2%)	233 (8.0%)	194 (10.3%)	
		9 or more weeks	5 (12.5%)	12 (4.8%)	67 (6.8%)	214 (7.4%)	260 (13.7%)	

Note: * *p* < 0.05; *** *p* < 0.001, assessed using chi-squared tests.

**Table 3:** Association between participation in warm-up exercises and the occurrence of complications.

Variables		Occurrence of Complications
Participation in warm-up exercises	Not at all	0.933 (0.418–2.083), *p* = 0.866
	Not really	1.650 (1.191–2.287), *p* = 0.003 **
	Neutral	1.319 (1.075–1.620), *p* = 0.008 **
	Somewhat	1.112 (0.950–1.302), *p* = 0.185
	Very much	1.000 (reference)

Note: ** *p* < 0.01, assessed using multivariate logistic regression analysis adjusted for athlete type, sex, age, frequency of sports participation, participation in a sports club, and injury recurrence.

**Table 4:** Association between participation in warm-up exercises and subsequent injury frequency.

Variables		Subsequent Injury Frequency			
		1	2	3	4
Participation in warm-up exercises (reference group: 5 or more times)	Not at all	0.759 (0.293–1.967), *p* = 0.571	0.805 (0.303–2.140), *p* = 0.663	0.619 (0.192–1.993), *p* = 0.422	0.928 (0.188–4.580), *p* = 0.926
	Not really	1.132 (0.717–1.788), *p* = 0.594	1.162 (0.729–1.855), *p* = 0.528	0.999 (0.592–1.685), *p* = 0.996	0.750 (0.314–1.793), *p* = 0.518
	Neutral	1.330 (1.007–1.758), *p* = 0.045 *	1.441 (1.085–1.914), *p* = 0.012 **	1.221 (0.892–1.672), *p* = 0.212	1.322 (0.848–2.061), *p* = 0.218
	Somewhat	1.332 (1.096–1.619), *p* = 0.004 **	1.406 (1.151–1.717), *p* = 0.001 *	1.173 (0.940–1.462), *p* = 0.157	1.074 (0.782–1.474), *p* = 0.659
	Very much	1.000 (reference)	1.000 (reference)	1.000 (reference)	1.000 (reference)

Note: * *p* < 0.05; ** *p* < 0.01, assessed using multivariate logistic regression analysis adjusted for athlete type, sex, age, frequency of sports participation, participation in a sports club, and injury recurrence.

**Table 5:** Association between participation in warm-up exercises and recovery duration.

Variables		Recovery Duration				
		Not Much Time; Resumed Activities Immediately	Less Than a Week	1–2 Weeks	3–4 Weeks	5–8 Weeks
Participation in warm-up exercises (reference group: 9 or more weeks)	Not at all	1.389 (0.411–4.694), *p* = 0.597	1.343 (0.407–4.432), *p* = 0.628	0.661 (0.204–2.140), *p* = 0.490	0.381 (0.098–1.471), *p* = 0.161	1.530 (0.468–4.998), *p* = 0.481
	Not really	3.560 (1.743–7.272), *p* < 0.001 ***	4.464 (2.243–8.885), *p* < 0.001 ***	2.743 (1.419–5.305), *p* = 0.003 **	2.193 (1.121–4.292), *p* = 0.022 *	1.948 (0.910–4.170), *p* = 0.086
	Neutral	1.940 (1.323–2.843), *p* = 0.001 **	2.639 (1.824–3.816), *p* < 0.001 ***	2.010 (1.432–2.823), *p* < 0.001 ***	1.530 (1.079–2.167), *p* = 0.017 *	1.188 (0.785–1.798), *p* = 0.415
	Somewhat	1.766 (1.350–2.310), *p* < 0.001 ***	1.694 (1.291–2.221), *p* < 0.001 ***	1.834 (1.440–2.335), *p* < 0.001 ***	1.634 (1.278–2.089), *p* < 0.001 ***	1.227 (0.918–1.641), *p* = 0.168
	Very much	1.000 (reference)	1.000 (reference)	1.000 (reference)	1.000 (reference)	1.000 (reference)

Note: * *p* < 0.05; ** *p* < 0.01; *** *p* < 0.001, assessed using multivariate logistic regression analysis adjusted for athlete type, sex, age, frequency of sports participation, participation in a sports club, and injury recurrence.

## Data Availability

The data presented in this study are available at https://www.sportsafety.or.kr/front/board/boardContentsListPage.do?board_id=42 (accessed on 1 January 2026).

## Abbreviations

The following abbreviations are used in this manuscript:

OR	Odds ratio
CI	Confidence interval
